# Patterns of Orchid Diversity and Their Potential Habitat Under Climate Change in Chongqing, China

**DOI:** 10.3390/biology15040351

**Published:** 2026-02-18

**Authors:** Huan Zhang, Mingwei Tang, Yiyun Wang, Rui Pan, Hongping Deng

**Affiliations:** School of Life Sciences, Southwest University, Beibei District, Chongqing 400715, China

**Keywords:** orchids, climate change, distribution pattern, MaxEnt, suitable habitat

## Abstract

Global climate problems and the sharp decline in biodiversity have attracted widespread attention. Orchids, as the “flagship” species of biodiversity, are important indicators of ecological changes. This study took Chongqing as the study area and conducted a comprehensive survey of orchids through field investigation combined with data review to clarify Chongqing’s diversity distribution pattern. The distribution of orchids was characterized by “high in the east and low in the west, high in the north and low in the south” horizontally. Vertically, the distribution was characterized by an obvious “unimodal distribution”, with higher abundance in the low and middle altitude areas of 500–1499 m. The minimum temperature of the coldest month (Bio6), isothermality (Bio3), altitude (Bio20), and precipitation of the wettest season (Bio16) were the main environmental factors affecting the distribution of the orchid habitat. The suitable habitat of orchids would be greatly reduced in the future (2070SSP-585), and the suitable habitat tends to migrate to the high-altitude areas; therefore, we should pay more attention to the conservation and sustainable use of orchid plant resources.

## Simple Summary

Orchids hold a pivotal position as climate change indicators due to their highly specialized biological traits. As a typical mountainous megacity, Chongqing is undergoing profound climatic changes, which have a rich diversity of orchids, but their distribution patterns and future changes under climate change remain unclear. Therefore, clarifying the distribution patterns of orchids in Chongqing and identifying the most suitable distribution areas under the current climate is particularly important.

## 1. Introduction

**Figure 1:**
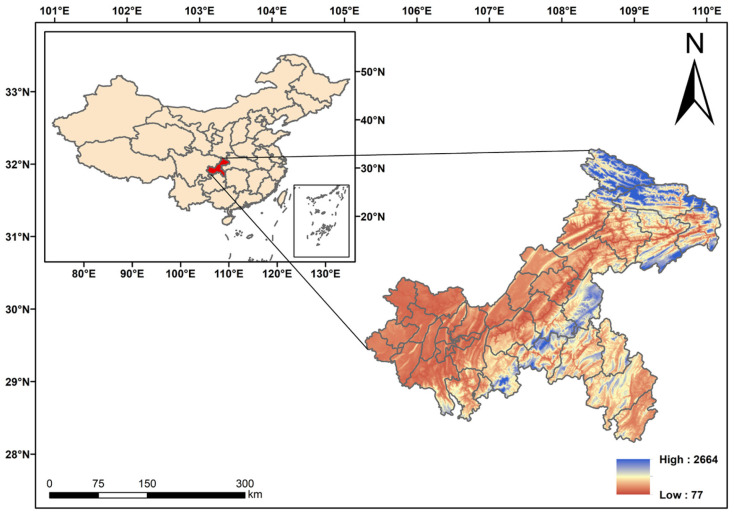
Map of the study area (High and Low indicate the altitude).

In recent decades, the global warming trend has become more pronounced. According to the Sixth Assessment Report (AR6) of the Intergovernmental Panel on Climate Change (IPCC), the global temperature is expected to rise by 1.5 °C between 2021 and 2040 [1]. The rapid changes in the climate are seriously affecting the distribution of species and may even lead to the loss of global biodiversity and the extinction of species [2]. Global biodiversity loss and species extinction, which will destroy ecosystems critical to environmental functioning and human health, have been major environmental issues in recent years [3]. In terms of the number of species, Orchidaceae is one of the largest families of flowering plants in the world, with about 744 genera and 31,072 species globally, and about 200 genera and more than 1600 species have been reported in China [4,5,6]. Globally, orchids are widely distributed, covering all continents except extreme arid deserts and polar regions. Species diversity hotspots are concentrated in tropical mountainous areas, such as Costa Rica, Panama, Colombia, Ecuador, and New Guinea [7]. In China, orchids are mainly found in the southwestern region, especially in the eastern part of the Himalayas, the Xishuangbanna region, the Hengduan Mountains region, and the eastern part of Taiwan [8,9]. Orchids are a highly evolved group of angiosperms and are of great scientific value, as well as having certain medicinal and ornamental properties [10,11]. Orchids are often referred to as the “flagship” species of biodiversity, i.e., their diversity reflects the plant diversity of a given region, and their abundance is directly proportional to the abundance of the local flora [12]. Orchids hold a pivotal position as climate change indicators due to their highly specialized biological traits. Firstly, their tiny seeds lacking endosperm require specific mycorrhizal fungi for both germination and seedling development—a symbiotic relationship that persists throughout their lives with remarkable specificity [13]. Secondly, orchids depend on intricate and often deceptive pollination systems, with many species relying exclusively on one or a few insect species. These tightly coupled, mutually exclusive relationships with fungi and pollinators make orchids particularly vulnerable to disruptions caused by climate-induced habitat shifts, phenological misalignment, or mismatched symbiotic partners. As a result, compared to plants with broader symbiotic networks, orchids respond to climate change more rapidly and intensely, allowing their distribution patterns and survival status to serve as early warning indicators of ecosystem changes [13,14,15]. Due to their stringent requirements for habitat, pollinators, germination conditions, and soil microorganisms [16,17], orchids also face threats such as climate change, habitat loss, and illegal harvesting and trade. As a result, orchids have become one of the most critically endangered plant groups [8,18]. In many biodiversity hotspots, the baseline species composition and population dynamics remain unclear, leading to limited or reduced conservation success. Therefore, the investigation of orchid diversity is of great significance to the understanding of local species diversity. Chongqing is located in the eastern part of Southwest China (Figure 1), with a typical subtropical monsoon humid climate, the transition zone of China’s northern and southern plants, and the core zone of the “East Sichuan-West Hubei Center”, one of China’s three major centers of endemic phenomena [19]. The geological landscape is complex, with numerous mountains and hills and high environmental heterogeneity, and it has rich and diverse species [20]. As a typical mountainous megacity, Chongqing is undergoing profound climatic changes. Recent studies indicate that under the combined impacts of global warming and rapid urbanization, the urban heat island effect in Chongqing will continue to intensify under future scenarios [21]. This unique dynamic of the mountain thermal environment constitutes a severe stress affecting the survival of local species. Chongqing is situated in a region that serves as a hotspot for the distribution of numerous orchid species, which are highly sensitive to climate change. However, when focusing on Chongqing, this unique mountainous municipality, existing biodiversity research exhibits significant gaps. On one hand, studies on local orchid species in Chongqing predominantly concentrate on taxonomic classification, population ecology, or genetic structure analysis of specific species. While these studies provide crucial foundations for understanding local orchid resources, their perspectives remain relatively limited [22]. Recent studies on Asian endemic orchids (e.g., the genus *Holcoglossum* Schltr.) have demonstrated that both widespread and endemic species may experience significant contraction or migration of their potential distribution ranges under future climate scenarios [23]. However, systematic assessments of how orchid diversity in Chongqing responds to the climate projections remain insufficient. Therefore, as the impacts of climate change become increasingly evident, utilizing the MaxEnt model and integrating future climate data to predict the potential distribution of orchids in Chongqing can not only provide critical scientific evidence for regional biodiversity conservation but also offer an important case study for understanding plant response patterns in complex mountainous environments.

Species Distribution Models (SDMs) are important tools to quantify species–environment relationships and predict potential geographic distribution by integrating species distribution point data with environmental variables. The core assumption is that species distribution is limited by environmental conditions and that existing distribution sites reflect their ecological needs [24]. The MaxEnt model is a statistical model based on maximum entropy theory, which analyzes and integrates incomplete data by using machine learning and maximum entropy to draw inferences or predictions, and it can predict the actual and potential distribution areas of species based on environmental variables and species distribution data [25]. The MaxEnt model can accurately predict suitable ranges with a small amount of geographic distribution data combined with environmental variables. The model is characterized by a small sample size, low sample bias, and high simulation accuracy over a small area [26]. However, the default parameters of the MaxEnt model may lead to overfitting. Therefore, in practical applications, an optimized MaxEnt model is often used to predict the future potential distribution of species. By using the R package ENMeval to fine-tune and evaluate key parameters (such as regularization factors and feature combinations), the potential overfitting issues caused by default parameters can be avoided. Ultimately, the optimal parameter combination is selected for final predictions based on validation metrics (e.g., AIC) [27,28,29]. The optimized MaxEnt model has been widely used in recent decades to study the impacts of climate change on species distribution, species richness, and distributional status, and the prediction of the potential distribution of species, and has played an important role in the applied research on endangered plants, medicinal plants, energy plants, and other highly valuable species [30,31].

This study clarified the current horizontal and vertical distribution patterns of orchids in Chongqing, and the suitable habitat under current and future climate conditions was evaluated by an optimized MaxEnt model. The objectives of this study include (1) to clarify the species, quantity, and distribution of Chongqing orchids; (2) to determine the optimal growth area of orchids in Chongqing under the current climate; and (3) to predict the suitable changes of orchids in Chongqing under future climate conditions. This will provide basic information and a reference basis for the development, utilization, and conservation of orchids in Chongqing.

## 2. Materials and Methods

### 2.1. Data Collection and Collation

**Figure 2:**
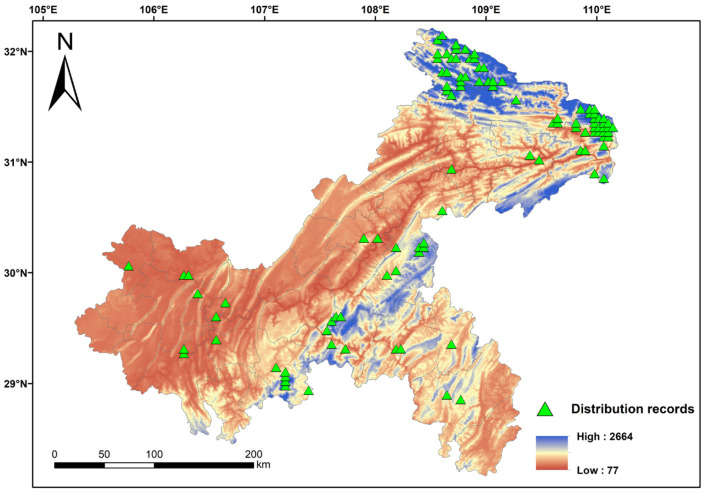
The 102 distribution records of orchids in Chongqing (High and Low indicate the altitude).

Through field surveys conducted in Chongqing from 2020 to 2023, combined with occurrence records of orchid species obtained from the National Plant Specimen Resource Center (NPSRC; https://www.cvh.ac.cn/ (accessed on 29 October 2024)) and the Global Biodiversity Information Facility (GBIF; https://www.gbif.org/ (accessed on 1 November 2024)), a total of 759 orchid distribution points (occurrence records) were obtained (Table S1). This study focuses on all orchids surveyed within the geographical scope of Chongqing. The orchids in Chongqing are mainly terrestrial type (genus *Bletilla* Rchb. f., *Goodyera* R. Br., and *Cymbidium* Sw., etc.), followed by epiphytic type (genus *Bulbophyllum* Thouars, *Dendrobium* Sw., and *Gastrochilus* D. Don, etc.), saprophyte type (genus *Gastrodia* R. Br., *Neottia* Guett, and *Corallorhiza* Gagnebin, etc.), semi-epiphyte type (*Cymbidium lancifolium* Hook. and *Pleione bulbocodioides* (Franch.) Rolfe), and subshrub type (*Galeola lindleyana* (Hook. f. & Thomson) Rchb. f. and *Galeola faberi* Rolfe) [3]. According to the altitude range of orchid plant distribution in Chongqing, the altitude range of orchid plant distribution was divided into five intervals with an altitude gradient of 500 m, i.e., 0–499 m, 500–999 m, 1000–1499 m, 1500–1999 m, and 2000–2499 m, to construct the vertical distribution pattern of orchids [32]. According to the principle of retaining one distribution point in a 5 × 5 km raster (The 5 km spatial dilution threshold ensures that only one distribution point remains in each environmental grid, effectively reducing environmental variable redundancy and sampling bias caused by spatial clustering of sampling points. This is a standard practice to improve model performance.) [33]. The ENMTools package (in R 4.4.2) was used to eliminate the wrong and duplicate error sites, and 102 sample points (unique grid localities) of the natural distribution of orchids were finally obtained (Figure 2) [34].

### 2.2. Environmental Data Acquisition and Processing

A total of 19 climate factors and 1 terrain factor were obtained from the World Clim (https://www.worldclim.org/ (accessed on 6 November 2024)) website, and the data were all utilized at 2.5 min spatial resolution. Climate data were included for the past (LGM, MH), the present (1970–2000), the 2050s (2041–2060 mean), and the 2070s (2061–2080 mean), and all data were exported to *.asc format [35]. The atmospheric circulation model for future climate data utilizes the BCC-CSM2-MR (Beijing Climate Center Climate System Model version 2-Medium Resolution) model [35]. Considering the influence of climate change scenarios on the results of model projections, three typical concentration pathways were selected for future climate scenarios, namely SSP-126, SSP-245, and SSP-585, which represent three greenhouse gas (GHG) emission scenarios: low, medium, and high, respectively [36].

The correlation coefficients of the 20 environmental factors were analyzed using the ENMTools package, and those with correlation coefficients less than |0.9| were retained [35,36]. Finally, 9 environmental factors were selected for modeling (Table S2).

### 2.3. MaxEnt Model Optimization

In this study, the MaxEnt model was optimized using the ENMeval package in R 4.4.2 [37]. The RM parameters were set from 0.5 to 4 with an interval of 0.5 for a total of 8 RM parameters. The Akaike Information Criterion Correction (AICc) was utilized to evaluate the fit and complexity of the model, and a training omission rate (OR10) of 10% was used to assess the degree of overfitting of the model. The parameter combination with the smallest delta. AICc was used as the best parameter to construct the model [38].

### 2.4. MaxEnt Model Running

To obtain the most predictive model and avoid overfitting, the maxent model was optimized using the ENMeval package in R 4.4.2. The RM parameter was set to 0.5–4 with an interval of 0.5, totaling 8 RM parameters. For the FC parameter, the maxent model provided five features: linear (L), quadratic (Q), hinge (H), product (P), and threshold (T). Six feature combinations were selected: L, LQ, H, LQH, LQHP, and LQHPT. The above 48 parameter combinations were tested using the ENMeval package. The Akaike Information Criterion Correction (AICc) was used to evaluate the fit and complexity of the models, while a 10% training omission rate (OR10) was employed to assess overfitting. Lower values indicated more accurate model predictions. The parameter combination with the smallest delta AICc was selected as the optimal parameter for constructing the model [39]. Simulations were performed by combining selected distribution information with selected environmental factors using MaxEnt 3.4.4 software. The RM parameter was set to 2.5, the FC parameter to LQHP, the maximum number of background points to 10,000, and the number of repetitions to 10. The Jackknife method in the MaxEnt software was utilized to calculate the contribution of each environmental variable. The accuracy of the model was evaluated by using the receiver operating characteristic curve (ROC) and the area under the ROC curve (AUC), with the AUC value between 0 and 1 [40]. The model results were selected as Logistic output and saved in *.asc format.

### 2.5. Compatible Grade Division

Currently, the methods of dividing the habitat suitability class include the natural discontinuity point method, the threshold value method, and the expert experience method. Generally, the habitat suitability class is divided into 3–4 classes [41,42]. The habitat suitability of a species is generally expressed as a value between 0 and 1. In this study, ArcGIS 10.8 software was utilized to divide and visualize the habitat suitability zones of orchids, and the habitat suitability index of orchids was classified using the natural discontinuities method to classify the potential habitats of orchids into unsuitable habitat (0–0.1), low suitability habitat (0.1–0.3), medium suitability habitat (0.3–0.5), and high suitability habitat (0.5–1).

## 3. Results

### 3.1. Geographical Distribution Pattern of Orchids

#### 3.1.1. Horizontal Richness and Distribution Characteristics

**Figure 3:**
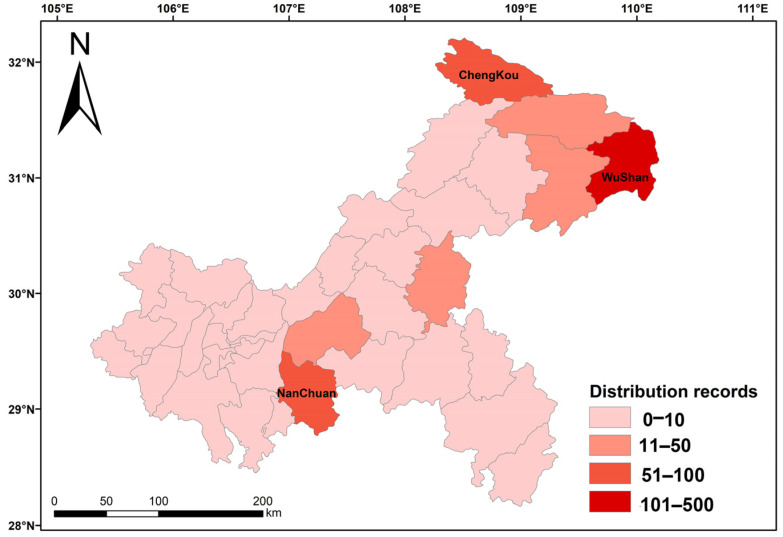
Horizontal distribution characteristics of orchids.

Orchid richness in Chongqing showed an overall distribution pattern of “high in the east, low in the west, high in the north, and low in the south” in the horizontal direction (Figure 3), with the highest orchid richness in the northeastern part of the city. From the perspective of districts and counties, Wushan County had the highest orchid richness (422 occurrence records), followed by Nanchuan District (97 occurrence records) and Chengkou County (96 occurrence records), whereas fewer orchids were found in the other districts and counties.

#### 3.1.2. Vertical Richness and Distribution Characteristics

**Figure 4:**
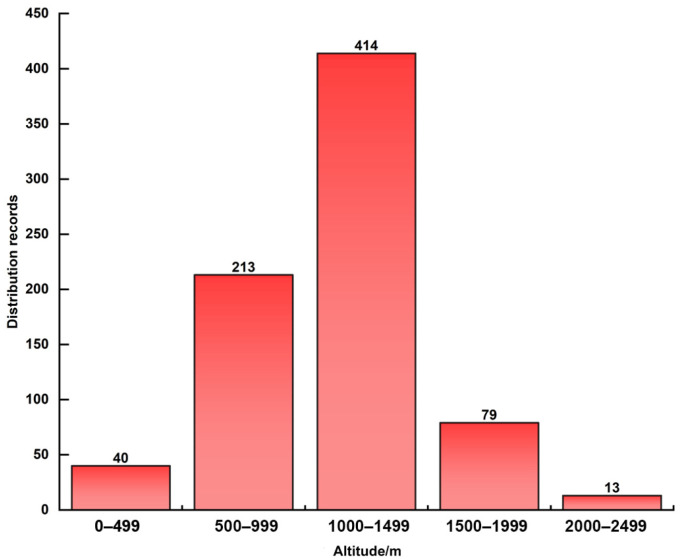
Vertical distribution characteristics of orchids.

From the viewpoint of the richness of orchid distribution in each altitude section, fewer orchids were in the low-altitude section below 500 m, and the high-altitude section above 2000 m. Orchids were mainly distributed in the altitude of 500–1999 m (observed abundance peaks at altitudes). The abundance of orchids in the vertical direction in Chongqing showed an obvious “unimodal distribution”, i.e., the abundance increased rapidly with the increase in altitude, reached the peak at 1000–1499 m, and then decreased slowly with the increase in altitude (Figure 4). Overall, the orchids in Chongqing were mainly distributed in the middle and low altitudes of 500–1499 m, with 627 occurrence records, accounting for 82.61% of the total number of occurrence records.

### 3.2. Prediction of Suitable Habitat for Orchids Under Climate Change Scenarios

#### 3.2.1. Model Optimization and Accuracy Assessment

**Figure 5:**
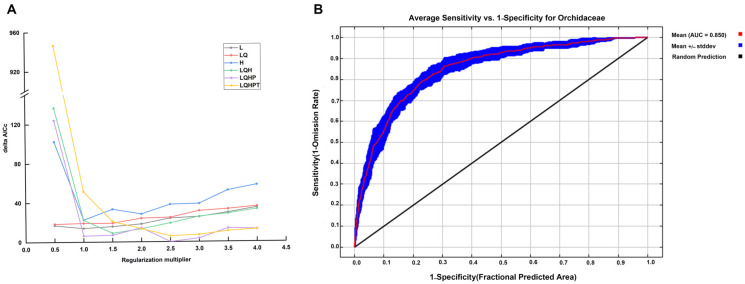
Model optimization and ROC curve prediction. (**A**) Delta AICc for orchids from the MaxEnt model under different parameter combinations, (**B**) the mean training AUC of the model.

According to the model optimization results, when FC was LQHP and RM was 2.5, the delta AICc value was 0. For the default parameters, FC is LQHPT and RM is 1.0, the delta AICc value is 51.33 (Figure 5A). When the model was optimal, the 10% training omission value was lower than that of the model with default parameters (Table 1). RM = 2.5 and FC = LQHP were chosen as the ideal model parameters in this study. Under this parameter setting, the MaxEnt model was used to predict the potential suitability habitat of orchids, and the average training AUC value of the model for 10 repetitions was 0.85 (standard error 0.02), indicating that the model had good prediction results (Figure 5B).

#### 3.2.2. Main Environmental Variables

In this study, 9 environmental factors including Bio3, Bio6, Bio12, Bio13, Bio14, Bio15, Bio16, Bio18, and Bio20 were screened for model prediction. Bio6, Bio3, Bio20, and Bio16 were the main environmental factors affecting the current distribution of orchid plant fitness zones, with a total contribution of 84.3%, with the coldest month’s minimum temperature having the greatest effect on the geographic distribution of orchids (Table 2).

**Figure 6:**
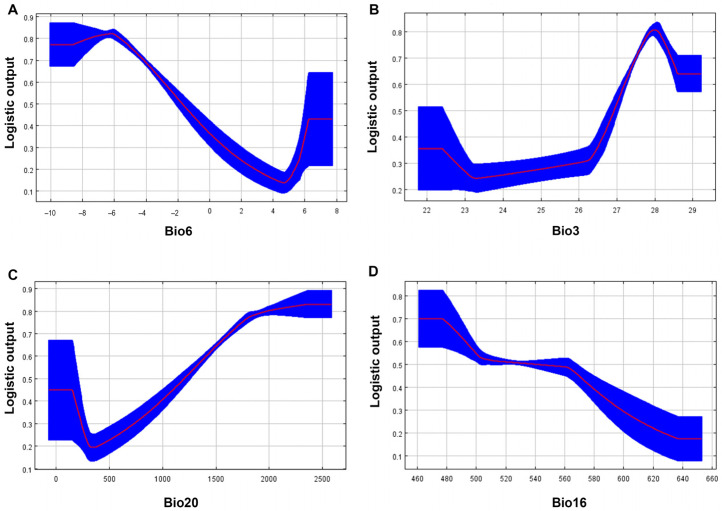
Response curves for the key environmental variables in the MaxEnt model. (**A**) Minimum temperature of the coldest month, (**B**) isothermality, (**C**) altitude, (**D**) precipitation of the wettest quarter.

The temperature had the greatest influence on the geographic distribution of orchids, with a decreasing trend in the presence probability as the minimum temperature of the coldest month increased. When the minimum temperature of the coldest month was −6 °C, the highest probability was 0.82, which was most suitable for the growth of orchids (Figure 6A). When the range of isothermality values was 27–29, the probability was >0.5, which was most suitable for orchids (Figure 6B). When the range of altitude was >1200 m, the survival probability of orchids was >0.5, indicating that the optimum growth altitude for orchids is 1200–2500 m (the optimal altitude range of climate predicted by the model) (Figure 6C). The probability of the existence of orchids decreased with the increase in precipitation in the wettest season, and the probability of existence was >0.5 when the precipitation was 461–540 mm, which was the most suitable for the growth of orchids, and followed the growth habit of orchids that prefer humidity and fear waterlogging (Figure 6D).

#### 3.2.3. Potential Geographic Distribution of Orchids in the Past Period

**Figure 7:**
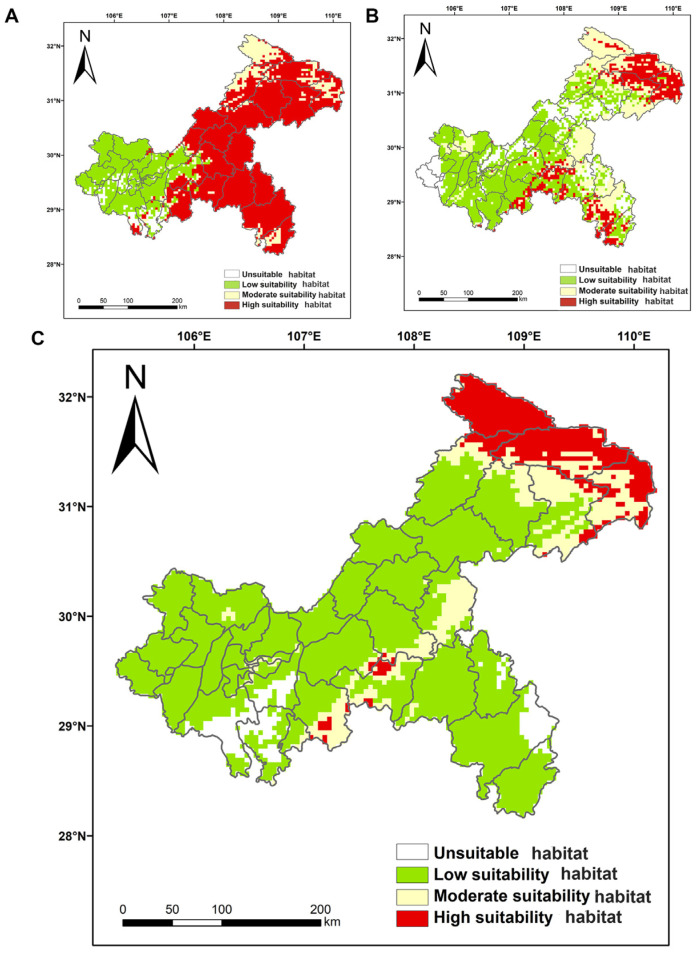
The potential suitability habitat of orchids in the paleo scenario and the current scenario in Chongqing. (**A**) Last Glacial Maximum, (**B**) mid-Holocene, (**C**) current scenario. Red represents the high suitability habitat, yellow represents the moderate suitability habitat, green represents the low suitability habitat, and white represents the unsuitable habitat.

The total area of the orchids’ habitat during the last glacial period was 79,570.74 km^2^, accounting for 96.57% of the total area of Chongqing. The high suitability habitat was the largest, with an area of 49,743.24 km^2^, accounting for 60.37%, and was mainly concentrated in the central and eastern parts of Chongqing. The moderate suitability habitat was scattered, with an area of 9800.7181 km^2^, accounting for 11.89%. The low suitability habitat was 20,026.75 km^2^, accounting for 24.30%, and was mainly distributed in the western part of Chongqing (Figure 7A). In the middle Holocene, the total area of orchids was 71,526.75 km^2^, accounting for 86.80%, of which 12,278.64 km^2^, accounting for 14.90% of the total area of the high suitability habitat, was mainly concentrated in Wuxi County, Wushan County, Fengjie County, and other districts and counties in the northeast part of Chongqing. The area of the moderate suitability habitat was 22,782.05 km^2^, accounting for 27.65%, and is scattered throughout the city. The low suitability habitat had the largest area of 36,466.07 km^2^, accounting for 44.25% (Figure 7B). The area of the high suitability habitat for orchids decreased greatly in the middle Holocene compared with the Last Glacial Maximum (LGM).

#### 3.2.4. Potential Geographic Distribution of Orchids During the Current Period

The prediction results showed that the total area of orchids’ suitability habitat in the current period is 77,906.46 km^2^, accounting for 94.55% of the total area of Chongqing, of which the high suitability habitat covered an area of 9615.80 km^2^, accounting for 11.67%, and was mainly concentrated in the northeast of Chongqing, Chengkou County, Wuxi County, Wushan County, and also in Kaizhou County and Fengjie County, Wulong County, and Nanchuan District had a small portion of high suitability habitat. The moderate suitability habitat was mainly distributed in the central and northeastern part of Chongqing, with an area of 11,575.94 km^2^, accounting for 14.05%. The low suitability habitat had the largest area of 56,714.72 km^2^, accounting for 68.83% (Figure 7C). Compared with the mid-Holocene and the last glacial period, the low suitable habitat of orchids in the current period has been greatly reduced, and its range has shifted from the central to the northeastern part of Chongqing.

#### 3.2.5. Potential Geographic Distribution of Orchids in the Future Period

**Figure 8:**
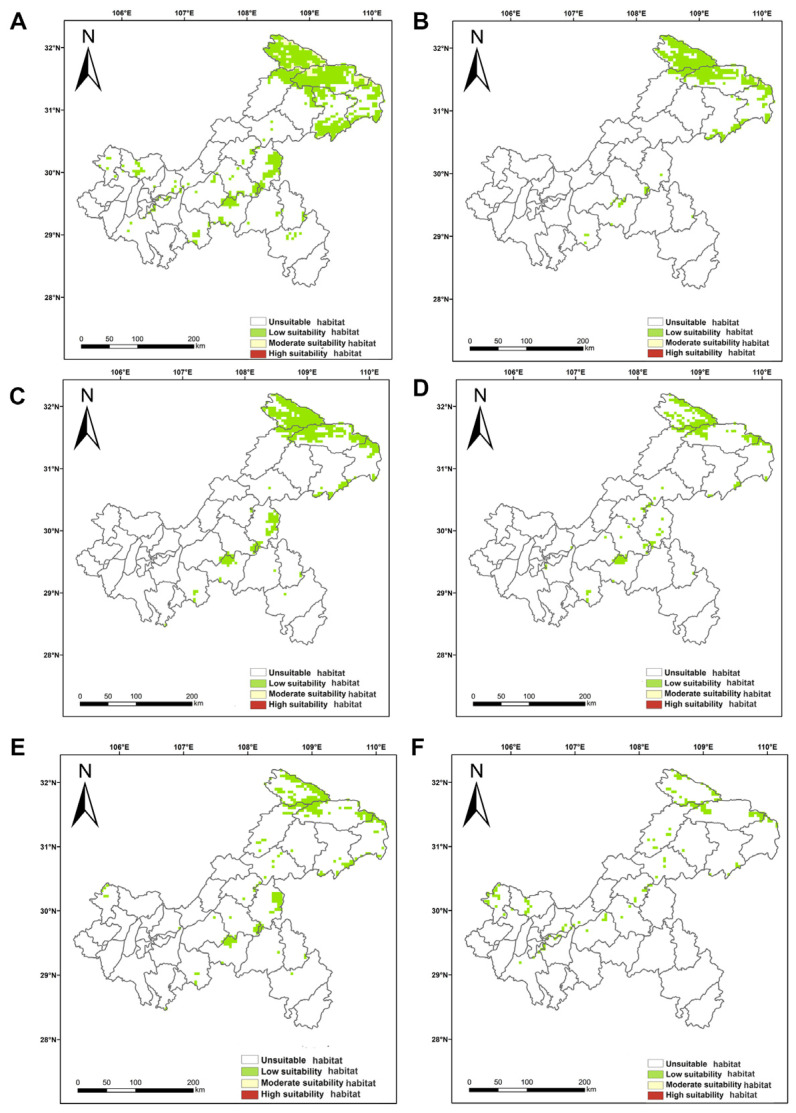
The potential suitability habitat of orchids in future scenarios in Chongqing. (**A**) 2050SSP-126, (**B**) 2070SSP-126, (**C**) 2050SSP-245, (**D**) 2070SSP-245, (**E**) 2050SSP-585, (**F**) 2070SSP-585. Red represents the high suitability habitat, yellow represents the moderate suitability habitat, green represents the low suitability habitat, and white represents the unsuitable habitat.

Under the 6 future climate scenarios, the high suitability habitat of orchids in Chongqing had completely disappeared, and the suitability habitat was mainly concentrated in the northeastern part of Chongqing and scattered in the central part of Chongqing. Under the SSP-126 scenario, the predicted suitability habitat of orchids in 2050 and 2070 was only the low suitability habitat and very little moderate suitability habitat (Figure 8A,B), while under the SSP-245 and SSP-585 scenarios, only the low suitability habitat was predicted (Figure 8C–F). The area of orchids’ suitability habitat under the SSP-126 scenario in 2050 was the largest, which was 15,126.3914 km^2^, accounting for 18.36%. Among them, the area of the MEDIUM suitability habitat was only 1479.3537 km^2^, accounting for 1.80% (Figure 8A). Under the SSP-585 scenario in 2070, the suitability habitat of orchids was the smallest, with an area of 2755.2962 km^2^, accounting for 3.34%, and all of them were low suitability habitat (Figure 8F).

**Figure 9:**
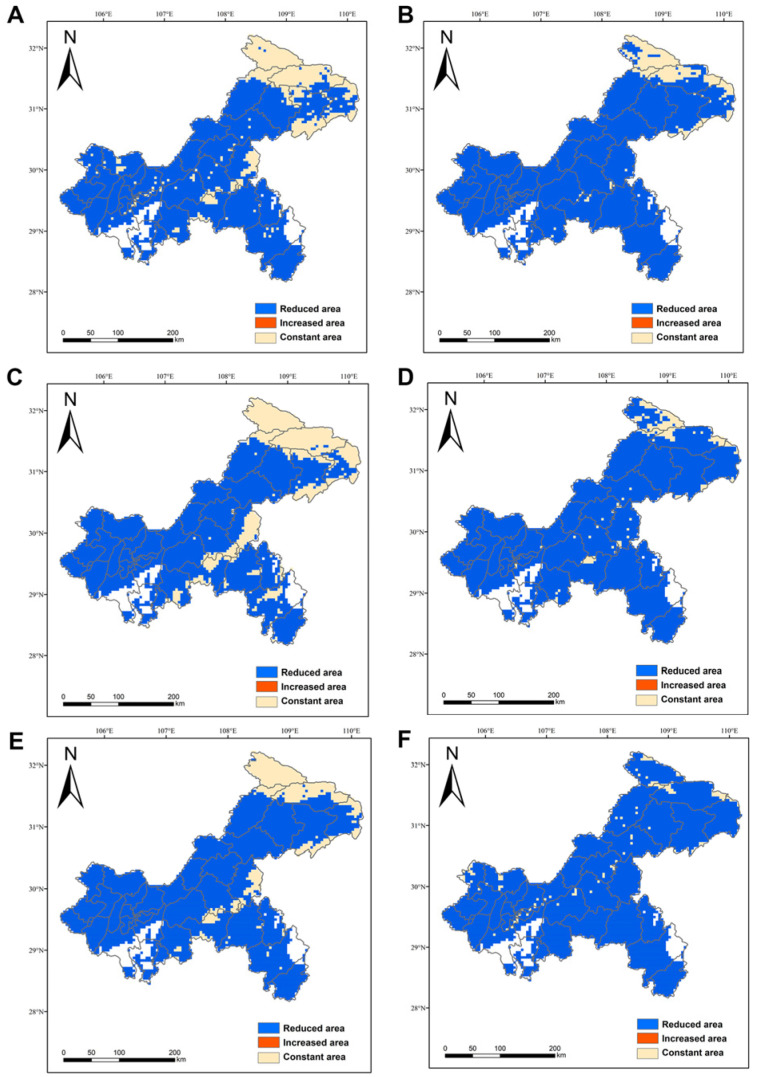
Changes in orchids in the adaptable region of Chongqing under the background of climate change. (**A**) 2050SSP-126, (**B**) 2070SSP-126, (**C**) 2050SSP-245, (**D**) 2070SSP-245, (**E**) 2050SSP-585, (**F**) 2070SSP-585. Blue represents the reduced area, red represents the increased area, and amber represents the constant area.

The model predicted that the area of the suitability habitat of orchids in the future period had shrunk considerably compared to the current period (Figure 9). The SSP-126 scenario in 2050 showed the smallest reduction in suitable habitat, 62,780.0718 km^2^, with a reduction of 80.58% (Figure 9A). In 2070, the SSP-585 scenario had the largest shrinkage of the suitability habitat, which was 75,151.1670 km^2^, with a shrinkage of 96.46% (Figure 9F). In terms of the degree of greenhouse gas emission, the higher the degree of greenhouse gas emission, the smaller the area of the suitable habitat of orchids, and the larger the shrinkage. In terms of the temporal change, the area of the suitability habitat of orchids was also decreasing with the increase in time, and the larger the shrinkage was (Figure 8 and Figure 9).

## 4. Discussion

### 4.1. Geographical Distribution Pattern of Orchids

Altitude is one of the key factors influencing the diversity and distribution patterns of orchids, as environmental factors such as mean temperature, water vapor pressure, and precipitation vary with altitude [43,44]. In this study, the vertical abundance of orchids in Chongqing showed an obvious “unimodal distribution” and was mainly concentrated in the 500–1499 m altitude section. The vertical distribution of orchids in the neighboring province of Yunnan showed the “intermediate expansion type”, but the main distribution of altitude segments was different [45]. The air humidity in the middle and low-altitude areas is often higher than that in the high-altitude areas [46]. Coupled with the lower temperature in the high-altitude areas, it forms such a “unimodal distribution” pattern, which also confirms the growth habit of orchids that like temperature and humidity [47]. Orchids in Chongqing are mainly distributed in the middle and low-altitude areas with high population density, which may lead to a reduction in their diversity due to the disturbance of anthropogenic activities.

Patterns of plant diversity in a region can indirectly reflect the status of biodiversity richness in that region [48]. After analysis, the horizontal direction of orchids in Chongqing showed the distribution characteristics of “high in the east and low in the west, high in the north and low in the south”, which were mainly concentrated in Wushan, Chengkou, Nanchuan, and other districts and counties. The geographical distribution of orchids in Chongqing was narrow and uneven. The reason for this distribution pattern may be that Wushan, Chengkou, Nanchuan, and other districts and counties had established nature reserves, which have complex natural environments and are subject to less anthropogenic interference, while other areas have strong anthropogenic interference, such as medicinal herbs digging, livestock grazing, excessive trade, etc., which threaten orchids and result in the difference in orchids diversity among districts and counties in Chongqing [6,49]. The model-predicted future high-adaptability zones (1200–2500 m) in this study coincide with the high-altitude core areas of existing nature reserves in Chongqing, such as Wulipo and Daba Mountain. This finding holds significant conservation implications: Chongqing’s current nature reserve system, based on biodiversity hotspots, has already preemptively protected what may become the most critical climate refuges in the future. These reserves, particularly World Natural Heritage sites like Wulipo with their vast altitude gradients (175–2680 m), provide crucial’ three-dimensional buffer zones’ for orchid species to cope with climate change through their continuous altitude gradients and complex microhabitats. They not only buffer the direct stress of rising temperatures but also offer spatial and temporal opportunities for species upward migration, population adaptation, and maintenance of essential ecological interactions. Therefore, consolidating and strengthening the management of these existing reserves while enhancing their ecological connectivity should be the most prioritized and effective strategy to address climate change and safeguard orchid species in Chongqing and the Daba Mountain region from extinction risks.

### 4.2. Prediction of Suitability Habitat of Chongqing Orchids Under a Climate Change Scenario

MaxEnt model, a widely used species distribution model for predicting the spatial distribution of species, which can simplify the complexity of natural systems, has been recognized by many researchers [50,51] and has important applications in the fields of endangered plant conservation and invasive species control [34,39,52,53,54].

As far as the environmental factors are concerned, Bio6, Bio3, Bio20, and Bio16 were the four environmental factors with the highest contribution rates, among which the coldest month minimum temperature was the environmental factor with the greatest influence on the growth of orchids, accounting for 46.3% of the total contribution rate. These plants showed a significant cold-adaptation tendency. The response curves showed a decreasing trend in the probability of the presence of orchids as the minimum temperature of the coldest month increased. The dominant role of the coldest month’s minimum temperature can be attributed to the following mechanisms: Physiologically, low temperatures directly restrict plant growth by inhibiting cell division and elongation. Even when photosynthesis continues, this inhibition regulates resource allocation through the gibberellin/della protein signaling pathway, causing plants to prioritize carbohydrate accumulation over cell wall formation under cold conditions, thereby creating a physiological bottleneck for survival and reproduction. Ecologically, the coldest month represents an environmental filter that species must endure throughout the year. Only orchid plants with sufficient frost resistance or cold acclimatization capabilities can maintain basic metabolism and protect sensitive tissues during this period. Consequently, this factor more decisively determines species’ geographic boundaries than growth season conditions [55,56]. Meanwhile, the overall orchids in the study area reach their optimal state at approximately −6 °C in Bio6, indicating the adaptive characteristics of this group to cold winters. This adaptive trait may render them particularly sensitive to future winter warming (increased Bio6), which should be a key consideration in the development of conservation strategies. This finding is particularly significant. It indicates that the diversity of orchids in Chongqing is not accidental but rather the result of long-term adaptation to the cold and humid mountainous climate of the region. This also suggests that future increases in extreme winter temperatures (Bio6 value rise) may directly compress their ecological niche, posing stress to the populations. Furthermore, this study revealed a significant negative correlation between the distribution probability of orchids in the study area and the minimum temperature of the coldest month (Bio6), with the peak occurring at approximately −6 °C. This result, which appears to contradict the common knowledge that orchids are ‘thermophilic,’ can be reasonably explained by their unique reproductive biological characteristics. Many terrestrial orchids distributed in temperate and subtropical mountainous regions, such as the genera *Cymbidium* sw. and *Cypripedium* L. species, present in this study area, rely on a sustained period of low-temperature vernalization for their flowering process [57]. Winter temperatures ranging from 0 °C to 10 °C are crucial for inducing and promoting the development of their flower buds, and the absence of this condition would lead to reproductive failure. Therefore, the model results likely indicate that winter low temperatures are a decisive climatic factor in ensuring the completion of critical reproductive stages and maintaining population renewal for local orchids. This also implies that future climate warming leading to increased winter temperatures will directly interfere with their vernalization process, thereby posing a serious threat to their population renewal and long-term survival, further reinforcing the previous conclusion about the rapid loss of habitats. At an altitude of 1200–2500 m, the survival probability of orchid >0.5, and with the increase in altitude, the survival probability of orchid plants showed an upward trend. High altitudes can become a refuge for orchids in the face of climate change. Studies have used meta-analyses to estimate the shift of species distribution to higher elevations at a median rate of 11.0 m per decade [58]. With the increase in precipitation in the wettest season, the survival probability of orchids decreased, and when the precipitation was 461–540 mm, the survival probability of orchids was >0.5, which was the most suitable for the growth of orchids. The orchids in Chongqing are mainly terrestrial. The root system of terrestrial orchids is fleshy and is more sensitive to changes in soil moisture because it is fixed in the soil [34]. If the root system of orchid plants is exposed to high precipitation for a long time, the root system will rot due to insufficient oxygen, further inhibiting photosynthesis and growth [59].

In terms of time scale, the area of suitable habitat for orchids in Chongqing shows a trend of gradual reduction, which is in line with the pattern of climate change. In terms of the degree of greenhouse gas emission, under the scenario of higher greenhouse gas emission, the area of suitable habitat for orchids in Chongqing is smaller, and the shrinkage is larger. Under the high greenhouse gas emission scenario, global warming intensifies, and high temperature and drought events occur frequently in the southwest region [60]. Our prediction serves as a strong risk warning: if climate continues to follow the high-emission trajectory, the current suitable climatic range of orchids will experience unprecedented compression, even facing the extreme risk of ecological niche “unsustainability.” This aligns with the significant reduction in orchid distribution areas observed in many similar studies. Extreme climate change due to global warming will seriously affect the distribution pattern of orchids. At the regional scale, compared with the colder climate scenarios in the past, the suitable area for orchids under the current climate scenario is shifted to the northeast, and the suitable area is drastically reduced; under the future climate warming scenario, the suitable area for orchids maintains the trend of shifting to the northeast and shrinking. The prediction results show that the future suitable habitats for orchids in Chongqing are mainly in the northeast of the city, such as Chengkou, Wushan, Wuxi, and other districts and counties, which will also be the preferred places for the introduction and cultivation of orchids in the future. These places are mostly high-altitude areas, indicating that the potential suitable habitats of orchids tend to migrate to high-altitude areas. We hypothesize that this future widespread disappearance of orchid plant habitats at low altitudes is due to abnormally high temperatures. Orchids are thermophilic and humidity-loving species, and the abnormally high temperatures caused by global warming will limit their growth, which is what will lead to the future trend of orchid migration to higher altitudes. This study predicts a dramatic loss of suitable habitats for orchids in Chongqing (up to 96.46%), with the most suitable altitudes concentrated between 1200 and 2500 m. The findings reveal a pronounced ‘extinction elevator’ effect: driven by climate warming, species are forced to migrate to higher elevations, but the geographical height of the Chongqing mountain range is limited (with a maximum of approximately 2796 m), and its migration space will soon be exhausted. Although local micro-landforms may provide temporary refuges, they cannot compensate for the overall collapse of suitable habitats at the macro scale. Consequently, orchid species in Chongqing, particularly those with high-altitude distribution and narrow ecological niches, face an extremely high risk of extinction due to the depletion of physical space. This necessitates forward-looking and urgent conservation actions. Orchids in Chongqing may migrate to higher altitudes in the future, which is consistent with the adaptive response of orchids under the background of global climate change. The results suggest that genus *Habenaria* Willd. and genus *Calanthe* R. Br. species may move to the northern and western high-altitude regions due to differences in climatic adaptation characteristics such as underground storage organs and evergreen/deciduous habits, respectively [61]. This study reveals that orchid diversity in Chongqing exhibits a classic unimodal distribution pattern, with the peak occurring in mid-altitude regions. This pattern has been documented in multiple mountain ecosystems worldwide, such as the tropical mountains of New Guinea [43]. However, it must be emphasized that the ecological mechanisms underlying this similarity differ significantly. Orchids in New Guinea are predominantly epiphytic, with their distribution strongly dependent on the high-humidity microenvironment of forest canopies. In contrast, orchids in Chongqing are mainly terrestrial, and their distribution pattern may be driven by a combination of factors, including soil characteristics, understory light conditions, and winter cold requirements. This contrast highlights the necessity to consider specific life-history strategies and regional environmental contexts when understanding species distribution patterns [62,63,64,65]. As the temperature increases, climate diversity tends to decrease at low altitudes but increases at high altitudes. High altitudes are important, not only as guardians of different climate types, but also as potential refuge for flora and fauna [66]. Our research shows that orchids can track suitable climate niches through vertical migration in response to climate change. Vertical environmental gradients in mountain ecosystems (e.g., temperature, precipitation, habitat heterogeneity due to altitude changes) can mitigate the impacts of climate change on biodiversity, provide migration pathways and refuge for species, and thus buffer the negative effects of global warming.

It is noteworthy that this study employed a spatial dilution threshold of 5 km, reducing the sample size to 102. However, multiple studies have demonstrated that the Maxent model exhibits significant robustness for small sample sizes, particularly when the remaining samples represent the key environmental gradients of the species [67,68,69]. The remaining points are widely distributed in Chongqing, covering the main environmental gradient from low altitude to high altitude, which provides the basis for the model to capture the core niche requirements of the target species. Meanwhile, we acknowledge that spatial dilution may lead to the loss of local microhabitat information, particularly for highly specialized rare species [69]. Therefore, it is recommended to conduct validation through higher-resolution studies in the future. Surely, the model’s predicted current climate suitability zones exceed the actual known distribution of target orchids. This highlights a fundamental characteristic of the MaxEnt model: it simulates species’ potential climatic niches rather than their actual distributions. In real-world landscapes, species colonization and survival are strongly constrained by a range of non-climatic factors, including but not limited to direct human interference (e.g., habitat conversion and overexploitation); critical non-climatic biological factors (e.g., distribution of obligate symbiotic mycorrhizal fungi); local soil and topographic conditions; and seed dispersal limitations caused by habitat fragmentation. Therefore, the model results identify potential areas for future conservation and restoration efforts, but translating this potential into actual populations requires comprehensive management measures addressing these constraints [70]. Orchids are sensitive to environmental changes and limited by their conditions, which makes it difficult for them to reproduce and spread, and the results of the MaxEnt model predictions provide valuable information for their conservation management. Our results can be applied to various aspects of orchid conservation, such as identifying distribution sites that may exist but have not yet been detected, recognizing potential dispersal sites, and prioritizing areas for introduction and cultivation. In view of the current distribution status of orchids in Chongqing, it is suggested to carry out more detailed species-level surveys on orchids distributed in Chongqing.

### 4.3. Protection Advice

According to our prediction, the highly suitable habitats for orchids are mainly concentrated in Wushan, Wuxi, Chengkou, Kaizhou, Fengjie, Wulong, Nanchuan, and other districts and counties, and most of these districts and counties have established nature reserves, so it can be seen that in-situ conservation is the main position for the conservation of orchid diversity. Therefore, in situ conservation can be maximized in the following ways: First, implement top-priority in situ conservation and targeted management for current suitable habitats, with special emphasis on high-adaptability zones. Second, utilize high-resolution topographic and remote sensing data to identify and prioritize potential mountain microclimate refuges in areas predicted to experience severe habitat loss. Third, immediately initiate feasibility studies and pilot projects for ex situ conservation, germplasm preservation, and assisted migration of critical orchid species, serving as a contingency plan for worst-case scenarios. In addition, launch integrated conservation programs: combining climate adaptation, mycorrhizal conservation, habitat restoration and ex situ conservation to support species to use micro-hedges for natural adaptation or assisted migration, and the prediction of orchid habitat under climate change conditions in this study can provide a certain reference for the selection of sites for relocation conservation, which, together with artificial propagation and cultivation techniques, may be able to contribute to the conservation of orchids. From 2000 to 2020, China has made great achievements in orchid conservation [5], but there is still a long way to go in the conservation of orchids.

## 5. Conclusions

This study elucidated the distribution pattern of orchids in Chongqing through comprehensive surveys and MaxEnt modeling and further identified the most suitable distribution areas under current climatic conditions. The results showed that the richness of orchids in Chongqing is high, but their geographic distribution is narrow and regionally uneven, and the maximum richness of orchids is in the low-middle altitude region of 500–1499 m. Furthermore, we predicted the potentially suitable habitats for orchids in Chongqing. The results showed that temperature, altitude, and precipitation were important factors affecting the habitat suitability of orchids, with temperature being the most important environmental factor. In the future, the area suitable for orchids was drastically reduced, and their potential habitat tended to migrate to higher altitude areas. This suggests that we should pay more attention to the threats to the survival of orchids caused by human interference, habitat destruction, and climate change and do a good job of protecting orchids.

## Figures and Tables

**Table 1:** Evaluation results of the MaxEnt model with different parameter settings.

Model Evaluation	Feature Combination	Regularization Multiplier	Value of the Delta Akaike Information Criterion Corrected Akaike	10% Training Omission Rate
Default	LQHPT	1	51.33	0.16
Optimized	LQHP	2.5	0	0.13

**Table 2:** The contributions of environmental variables.

Code	Environmental Variables	Percent Contribution
Bio6	Min Temperature of Coldest Month/°C	46.3
Bio3	Isothermality (Bio2/Bio7) × 100	22.8
Bio20	Altitude/m	8.4
Bio16	Precipitation of Wettest Quarter/mm	6.8
Bio13	Precipitation of Wettest Month/mm	3.6
Bio14	Precipitation of Driest Month/mm	3.3
Bio15	Precipitation Seasonality (Coefficient of Variation)	3.1
Bio18	Precipitation of Warmest Quarter/mm	2.9
Bio12	Annual Precipitation/mm	2.8

## Data Availability

All data are included in the text.

## Supplementary Materials

The following supporting information can be downloaded at: https://www.mdpi.com/article/10.3390/biology15040351/s1, Table S1: Georeferenced occurrence records of Orchidaceae Table. Table S2: Bioclimatic variables.
